# Based on Systematic Pharmacology: Molecular Mechanism of Siwei Jianbu Decoction in Preventing Oxaliplatin-Induced Peripheral Neuropathy

**DOI:** 10.1155/2020/8880543

**Published:** 2020-10-06

**Authors:** Peng Zhang, Yuting Lu, Chao Yang, Qiuyan Zhang, Yangyan Qian, Jinshuai Suo, Peng Cheng, Jing Zhu

**Affiliations:** ^1^Jiangsu Key Laboratory for Pharmacology and Safety Evaluation of Chinese Materia Medica, Department of Pharmacy, Nanjing University of Chinese Medicine, Nanjing210023, China; ^2^Departments of Neurology and Neuroscience, Johns Hopkins University School of Medicine, Baltimore, MD, USA

## Abstract

Chemotherapy-induced peripheral neuropathy (CIPN) is a dose-limiting side effect caused by chemotherapy drugs, and its existence seriously affects the quality of life of patients. We first established an oxaliplatin-induced peripheral neuropathy (OIPN) model and then measured and evaluated mechanical hyperalgesia, thermal nociception, cold allodynia, and intraepidermal nerve fiber (IENF) density to determine Siwei Jianbu Decoction's role in preventing OIPN. Then, we conducted a systematic pharmacological study that revealed important roles for the MAPK signaling pathway and proinflammatory immune pathway and confirmed these roles by western blot, immunofluorescence, and qPCR. The data show that Siwei Jianbu Decoction can effectively prevent oxaliplatin-induced neuroinflammation by inhibiting an increase in NF-*κ*B expression via downregulation of p-ERK1/2 and p-p38. The present study showed that SWJB may be beneficial in preventing oxaliplatin-induced peripheral neuropathy.

## 1. Introduction

Currently, colorectal cancer has become a common type of cancer, and its present global incidence and mortality rate are among the highest. Oxaliplatin is an antitumor drug combined with 5-fluorouracil (5-FU) and leucovorin (LV), which is used as an adjuvant treatment for patients with colon cancer reoperation [1, 2]. However, severe peripheral neuropathy is induced in approximately 90% of patients during the course of treatment [3]. Common symptoms include allodynia, hyperalgesia, dysesthesia, and paranaesthesia [4]. These side effects limit the use of oxaliplatin. It has been shown that the activation of glial cells and the upregulation of proinflammatory cytokines play a crucial role in the occurrence and development of chemotherapy-induced neuropathic pain [5]. In particular, oxaliplatin induces neuropathic pain via astrocyte-activated cosignaling and the mitogen-activated protein kinase (MAPK) pathway [6, 7]. We know that the MAPK signaling system is activated by extracellular stimuli, leading to an intracellular response. These stimuli provide links between transmembrane signal transduction and transcriptional changes in different environmental signals, such as cytokines, growth factors, oxidative stress, and inflammation [8]. In our study, we found that inflammation may be related to nuclear factor kappa-B (NF-*κ*B) and extracellular regulated protein kinases (ERK/p38) MAPK signaling pathways in mouse models of oxaliplatin-induced peripheral neuropathic pain (OIPN). Similarly, studies have found that celecoxib reduces oxaliplatin-induced hyperalgesia by inhibiting ERK1/2 signaling in the spinal cord [9], and the major drugs used clinically for the prevention and treatment of OIPN, such as glutathione, vitamin E, amifolin, amantadine, mannitrite, and norepinephrine reuptake inhibitors, are not effective against neuroinflammation in OIPN [10, 11].

As a result of the overall concept of traditional Chinese medicine theory and the historical clinical practice of treating complex diseases, traditional Chinese medicine (TCM) is attracting attention worldwide. Siwei Jianbu Decoction is a well-known recipe created by Professor Huang Huang of Nanjing University of Chinese Medicine. In the following, J12 will be used as an abbreviation for Siwei Jianbu Decoction. This recipe is made up of four herbs: *Paeonia veitchii* Lynch, *Salvia miltiorrhiza* Bge, *Achyranthes bidentata* Blume, and *Dendrobium nobile* Lindl, and its main effect is to activate blood circulation, relieve stasis and pain, improve blood supply to the lower limbs, and restore lower limb function. J12 is commonly used in the treatment of diabetic peripheral neuritis, diabetic foot, lower extremity thrombosis, and other diseases [12]. However, the therapeutic mechanism of J12 is not clear, and there are few studies on the analgesic effect of J12 on OIPN.

Therefore, the purpose of our present study was to elucidate the roles of J12 in peripheral neuropathic pain caused by oxaliplatin and whether the MAPK and ERK/p38 signaling pathways of oxaliplatin are related to the preventive effect of J12 on OIPN. First, the oxaliplatin-induced neuropathy model was established. The OIPN prevention effect of J12 was then confirmed by measurement of mechanical hyperalgesia, measurement of thermal nociception, measurement of cold allodynia, and plantar nerve fiber (IENF) density determination. In addition, because of the complexity and diversity of traditional Chinese medicine formula ingredients, we adopted a systematic approach to study the possible mechanisms, and it was finally determined that the major pathways of MAPK and proinflammatory immunity play an important role. Based on the findings of systemic pharmacology, we also validated the prediction of systemic pharmacology on the mechanism of J12 prevention and treatment of OIPN by studying the expression of NF-*κ*B and proinflammatory factors (TNF-*α*, IL-1*β*, IL-6).

## 2. Materials and Methods

### 2.1. Reagents

Oxaliplatin (Sigma, USA) was dissolved in 5% glucose and formulated to a final concentration of 0.4 mg/mL. J12, containing four Chinese herbs, was purchased from Jiangsu Provincial Hospital (Nanjing, Jiangsu, China) and deposited at Nanjing University of Chinese Medicine. The component herbs were decocted twice, each for 1 h. The decoction was filtered, and the filtrates were combined and concentrated by rotary evaporation under reduced pressure to 120 mL, which is equivalent to 1 g/mL of the original drug. To ensure its quality and stability, we purchased different batches of raw medicinal materials, divided them into ten parts of the same specification, made the original liquid according to the same decoction method, and tested its fingerprint. The results are shown in the supplementary material (Figure S1-S2).

### 2.2. Animals

Male adult C57BL/6 mice between 6 and 8 weeks of age were purchased from Qinglong Mountain Animal Breeding Farm (Nanjing, China) and were kept under specific pathogen-free conditions with air conditioning and a 12 h light/dark cycle. The mice were housed in cages with free access to food and water at a constant temperature (20°C ± 2). The experimental protocols were approved by the Animal Committee of Nanjing University of Chinese Medicine. All experiments were tested in a blinded manner.

After one week of adaptation to the environment, thirty-two C57 mice were randomly divided into 4 groups as follows: normal control group (normal saline), OXA group (4 mg/kg of body weight), and O+J12 (H) and O+J12 (L) groups (daily dose 10 g/kg and 5 g/kg of body weight, respectively). The O+J12 (L, H) group was given J12 by oral enema for 6 weeks. From the third week, the OXA group and O+J12 (L, H) group received i.p. injections of 100 *μ*L/10 g oxaliplatin (Sigma, USA) solution (prepared with 5% glucose) on Mondays and Thursdays (1 hour after J12) for 4 weeks [13]. A total of 8 injections at a cumulative dose of 32 mg/kg of OHP were given. The dose of the J12 decoction was converted from Professor Huang Huang's outpatient medical records and combined with the equivalent dose conversion between humans and animals [12].

### 2.3. Animal Behavior Tests

Behavioral tests were performed on days 0, 7, 14, 21, 28, 35, and 42. Body weight was recorded before the beginning of each behavioral study. Measurements were repeated three times for each mouse, and the mean was the behavioral threshold for each mouse. The investigators were unaware of the treatment group.

#### 2.3.1. Measurement of Mechanical Hyperalgesia

A dynamic plantar aesthesiometer (DPA, Ugo Basile, Italy) was used to measure the mechanical retraction threshold of the mice. The mice were placed in a plexiglass box on a metal sieve for 30 minutes, and the tactometer was calibrated with a 0.5 mm filament. The intensity of the stimulus to the middle of the plantar foot of the mouse was increased (the setting frequency was 1 g/s, the maximum force was 10 g) while slowly increasing the stimulus pressure. When a foot withdrawal reaction occurs, the system records the latency time and the animal's foot withdrawal reaction pressure. Each mouse was repeatedly measured 3 times, and the average value was the mechanical withdrawal threshold of each mouse.

#### 2.3.2. Measurement of Thermal Nociception

A plantar stimulation pain meter (37370, Ugo Basile Plantar Test Apparatus, Italy) was used to measure the reflex threshold of heat pain and contraction. The mice were placed in a plexiglass box on a glass plate for 30 minutes. The response time to irradiation until the mice withdraw their feet was measured. To prevent the skin of the mice from being burned, the automatic cut-off time was set to 30 s. Each mouse was repeatedly measured 3 times, and the average value was the heat pain threshold of each mouse.

#### 2.3.3. Measurement of Cold Allodynia

Determination of cold pain threshold: the upper body of the mouse was fixed, and 1/3 of the tail tip was placed in an ice water bath at 4°C. The time from the tail tip entering the water to tail swing was recorded. To prevent the tail from frostbite, the cut-off time was set to 30 s. Each mouse was repeatedly measured 3 times, and the average value was the cold pain tail flick threshold of each mouse.

### 2.4. Intraepidermal Nerve Fiber (IENF) Density Determination

According to reports, the lack of IENFs plays a key role in neuropathy caused by various chemotherapy drugs (such as oxaliplatin) [14]. On the last day of the sixth week, the plantar skin tissues of the mice were removed, fixed in a periodate-lysine-paraformaldehyde (PLP) solution for 18-22 hours, and then transferred to a 30% sucrose-containing PBS solution to dehydrate until the tissue subsided, and frozen sections were made within one week. The tissue was embedded with optimal cutting temperature compound (OCT) and sectioned frozen (continuously cut perpendicular to the epidermis) with a thickness of approximately 30 *μ*m. The sections were subsequently stained, and the state of the epidermal nerve fibers was observed under an inverted microscope.

### 2.5. Acquisition of Compounds in J12

The compounds of four herbs in J12 were gathered from the Traditional Chinese Medicine Systems Pharmacology Database and Analysis Platform (TCMSP, https://tcmspw.com/tcmsp.php) and Shanghai Institute of Organic Chemistry of CAS, Chemistry Database [DB/OL] (http://www.organchem.csdb.cn**)** for further screening [15].

#### 2.5.1. Screening of Parameters of Active Compounds

J12 is composed of four medicinal herbs, including Achyranthes bidentata, Akabane, Salvia miltiorrhiza, and Dendrobium. Therefore, we combined drug half-life (t1/2) (HL), drug-likeness (DL), oral bioavailability (OB), and blood–brain barrier (BBB) screening with Caco-2 permeability (Caco-2) evaluation to identify the active compounds in J12 [16].

Ultimately, we screened the active compounds by setting the following parameters: HL ≥ 4, DL ≥ 0.18, OB ≥ 30%, BBB ≥ −0.3, and Caco-2 ≥ −0.4 [17].

#### 2.5.2. Drug Target Prognostication for J12 and Construction of Compound–Target

To predict the potential targets of the active compounds in J12 screened above, we employed a systematic drug targeting method developed by Yu and others [18]: that is, we combined computer prediction model (combining two effective methods: support vector machine and random forest), SEA search tool (http://sea.bkslab.org/), and TCMSP database (http://lsp.nwu.edu.cn/tcmsp.php) and finally obtained the potential of the J12 target.

To gain a deeper understanding of the relationship between compounds and targets, we used Cytoscape (https://cytoscape.org/) software to build a composite target network.

#### 2.5.3. Recognition of OIPN Targets

To further analyze known targets associated with oxaliplatin-induced peripheral neuropathy, we searched five databases: DrugBank (http://www.drugbank.ca/), OMIM (http://www.omim.org/), GAD (http://geneticassociationdb.nih.gov/), PharmGKB (https://www.pharmgkb.org/index.jsp), and TTD (http://database.idrb.cqu.edu.cn/TTD/) [15]. We searched these databases using the keyword “oxaliplatin-induced peripheral neuropathy” and collected oxaliplatin-induced peripheral neuropathy-related targets.

#### 2.5.4. Construction of the Protein–Protein Interaction (PPI) Network and Recognition of Topological Features

An interactive network for potential J12 targets was constructed using the Cytoscape built-in plugin, Bisogenet, and Cytoscape software was used for visualization (version 3.7.1) [19]. In the same way, we built an interactive network for OIPN-related targets. The two networks were then combined to form a core protein-protein interaction (CPPI) network. To identify the necessary proteins in this PPI network, subsequent concentration analysis was performed using the Cytoscape plugin CytoNCA [20]. We filtered the major hubs of the network by the following six topology characteristics: “degree center (DC),” “intermediate center (BC),” “compact center (CC),” “feature vector center (EC),” “network center (NC),” and “local average connection (LAC).” These parameters are defined and computed in predefined formulas that represent the topological significance of the nodes in the network.

#### 2.5.5. Pathway Enrichment Analysis

We then applied the DAVID database (http://david.ncifcrf.gov) and the Metascape database (https://metascape.org/) for KEGG annotation of nodes in J12 and Omicshare Tools to construct visual enrichment results to analyze potential targets, signaling pathways, and related biological processes in the gene network that may be involved in the regulation of active components in J12.

### 2.6. Western Blotting

Extraction of total tissue protein: all operations were performed on ice. Mouse DRG tissue and sciatic nerve tissue were removed from the -80°C ultralow temperature refrigerator, and 500 *μ*L RIPA strong lysate and 5 *μ*L protease were added to the tissue of each mouse at a ratio of 100 : 1 inhibitor (PMSF) mixture. After grinding the homogenate, centrifugation was performed in a low temperature centrifuge at 12000 rpm and 4°C for 15 min, the supernatant was slowly removed, and the protein concentration was measured using a BCA kit (P0010; Beyotime, China) on a microplate reader (Infinite M200 Pro; Tecan, Switzerland). The proteins were analyzed by 8-12% SDS-PAGE gels and transferred to polyvinylidene fluoride membranes (Millipore, Billerica, MA). Nonspecific binding sites were blocked with 5% (*w*/*v*) BSA (4240GR100; BioFroxx), and the membranes were incubated with the primary antibodies NF-*κ*B, ERK1/2 (Abcam), phospho-p38, phospho-SAPK/JNK, phospho-ERK1/2, p38, SAPK/JNK, and GAPDH (Cell Signaling Tech). The membranes were washed 4 times in TBST and then incubated with the secondary antibodies for 1 h at room temperature on a shaker. Protein expression was measured by using an ECL system (UV200R; Tanon).

### 2.7. Immunofluorescence

At the end of week 6, L4 and L5 DRGs were taken from mice for immunohistochemical analysis. The sample tissue was placed in a paraformaldehyde fixative solution and placed at a temperature of 4°C. The tissue was then transferred to 20% sucrose for dehydration for 24 h, and then, the DRG was cut to a thickness of 10 *μ*m and mounted on a glass slide. Briefly, sections were blocked for 1 hour at room temperature in 5% normal goat serum and 0.5% Tween-20 in PBS and incubated overnight at 4°C with a primary antibody containing NF-*κ*B (1 : 1000; Abcam). The sections remained overnight at 4°C. After being washed three times with PBS, sections were incubated with FITC-conjugated secondary antibodies (1 : 100) in the dark at room temperature for 1 hour. After extensive washing, the sections were stained with DAPI. The immunostained DRGs were then observed under an inverted fluorescence microscope.

### 2.8. Real-Time Quantitative Polymerase Chain Reaction (PCR)

Total RNA was extracted from DRG tissue using a TRIzol extraction kit (Invitrogen, Carlsbad, USA). CDNA was synthesized using the reverse transcription kit TransStart Top Green qPCR SuperMix (TransGen Biotech, China). Real-time PCR was performed using an Applied Biosystems 7500 RealTime PCR System (Life Technologies, USA). Due to different species, qPCR-related primers were provided by Shanghai Shenggong Co., Ltd. The sequences are as follows:

GAPDH (forward: 5′ TTCCTACCCCCAATTATCCG 3′;

reverse: 5′ CATGAGGTCCACCACCCTGTT 3′).

TNF-*α* (forward: 5′ GCGACGTGGAACTGGCAGAAG 3′;

reverse: 5′ GAATGAGAAGAGGCTGAGACATAGGC 3′).

IL-6 (forward: 5′-AGACTTCCATCCAGTTGCC TTCTTG-3′

reverse: 5′-CATGTGTAATTAAGCCTCCGACTTGTG-3′).

IL-1*β* (forward: 5′ TTCAGGCAGGCAGTATCACTCATTG 3′;

reverse: 5′ ACACCAGCAGGTTATCATCATCATCC 3′).

NF-*κ*B (forward: 5′ CTGGTGCATTCTGACCTTGC 3′;

reverse: 5′ GGTCCATCTCCTTGGTCTGC 3′).

### 2.9. ELISA Measurements

Approximately 200 *μ*L of blood sample was collected from the eye socket of the mouse, and the supernatant of the blood sample was gathered after centrifugation at 3,000 × g for 15 minutes. According to the manufacturer's instructions, ELISA analysis kits (NF-*κ*B, MBE10044; TNF-*α*, MBE10037; IL-6, MBE10288; and IL-1*β*, MBE10289) were used to measure the concentration of IL-1*β*, NF-*κ*B, TNF-*α*, and IL-6 in the supernatant of the blood.

## 3. Statistical Analysis

All the experimental data are presented as the mean ± standard deviation (SD) and were analyzed with GraphPad Prism 8. The *p* values were tested by one-way analysis of variance (ANOVA) with the Tukey post hoc test for multiple comparisons. Data comparisons were considered to be significant if *p* < 0.05.

## 4. Results

### 4.1. J12 Prevents Oxaliplatin-Induced Hypersensitivity to Pain in Mice

**Figure 1:**
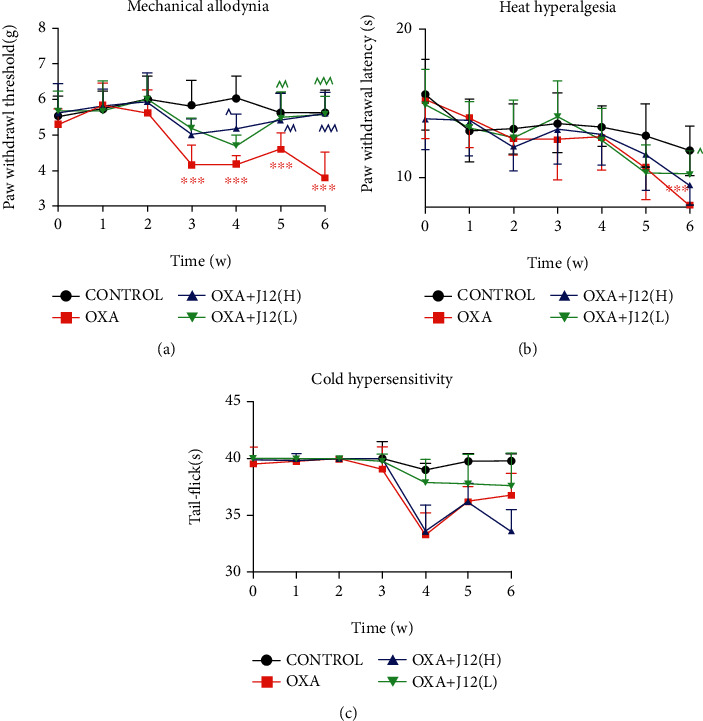
J12 treatment prevented OXA-induced inflammatory pain. J12 treatment prevented OXA-induced mechanical allodynia (a), heat hyperalgesia (b), and cold hypersensitivity (c). ^∗∗^ vs. Control, *p* < 0.01; ^∗∗∗^ vs. Control, *p* < 0.001; ^ vs. OXA, *p* < 0.05; ^^ vs. OXA, *p* < 0.01; ^^^ vs. OXA, *p* < 0.001, *α* = 0.05; *n* ≥ 6.

To verify whether J12 prevented oxaliplatin-induced peripheral neuropathy, we followed the behavioral experiment plan, as shown in Figure 1(a). There was a significant difference in the paw withdrawal threshold between the OXA group and the CONTROL group from the third week. In the line chart, J12 was effective from the third week by comparing the treatment groups and the OXA group. However, the preventive treatment groups and the OXA group showed significant differences in weeks 5 and 6 from the data analysis (Figure 1(a)).

The data on heat hyperalgesia of mice in each group showed significant differences in the sixth week. The OXA group mice were more sensitive than the control. Moreover, the difference between the OXA group and O+J12(L) group demonstrated that the prophylactic administration of J12 could attenuate oxaliplatin-induced heat hyperalgesia (Figure 1(b)).

The line chart shows subtle differences between the groups from the fourth week to the sixth week. The O+J12(L) group showed better preventive effects than the O+J12(H) group (Figure 1(c)). In summary, these results indicated that J12(L) successfully prevented OXA-induced neuropathic pain in mice.

### 4.2. J12 Prevented Oxaliplatin-Induced Loss of IENFs

**Figure 2:**
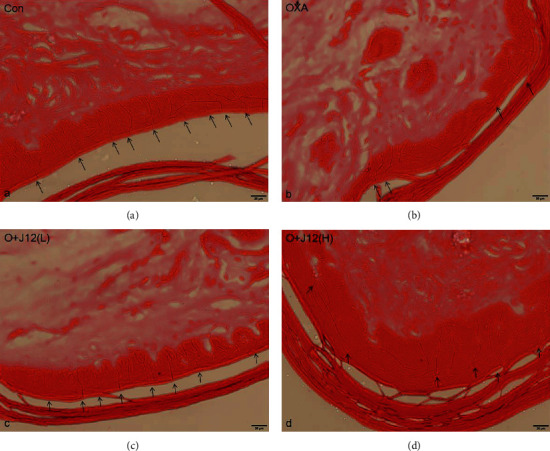
Animals were sectioned and stained with PGP9.5. Hindpaw skin tissue from control (a), OXA (b), O+J12 (L) (c), and O+J12 (H) (d) demonstrated that J12 could prevent oxaliplatin-induced loss of intraepidermal nerve fiber (IENF) density (scale bar = 20 *μ*m).

The immunofluorescence detection of PGP9.5 (a marker of IENFs) showed that compared with the control group (Figure 2(a)), the IENF density of the OXA group obviously decreased (Figure 2(b)). However, the prophylaxis groups reflected significant preventive effects, preventing the loss of epidermal innervation (Figures 2(c) and 2(d)). The results of our study demonstrated that J12 could prevent the loss of epidermal nerve fibers induced by oxaliplatin.

### 4.3. Systems Pharmacology Revealed Potential Therapeutic Effects of the MAPK Signaling Pathway in OIPN

J12 contains four herbs, but the mechanism of J12 treatment of OIPN remains unclear because of the multicomponent, multitarget nature of the herbal component. Therefore, we conducted a systematic approach to investigate the possible pharmacological mechanisms of J12.

A total of 471 compounds were collected from J12 to screen for active compounds by indicator, including drug half-life (HL), drug similarity (DL), oral bioavailability (OB), blood-brain barrier (BBB), and Coco-2 permeability (Coco-2) with the following criteria: HL ≥ 4, DL ≥ 0.18, OB% ≥ 30%, BBB ≥ -0.3, and Caco-2 ≥ -0.4 [17]. Finally, 64 potential compounds were screened. TCM compounding can play a therapeutic role through multiple targets. Therefore, we adopt a systematic approach to predict the potential targets of J12, and 88 potential targets were predicted: 49 for Salvia miltiorrhiza Bge, 73 for Achyranthes bidentata Blume, 62 for Paeonia veitchii Lynch, and 39 for Dendrobium nobile Lindl. It is worth noting that some herbs have similar targets; for example, four herbs can affect mitogen-activated protein kinase 14 (a key enzyme in the p38-MAPK signaling pathway), which is a key regulator of proinflammatory cytokine biosynthesis, making different components of the pathway a therapeutic potential target for autoimmune and inflammatory diseases.

**Figure 3:**
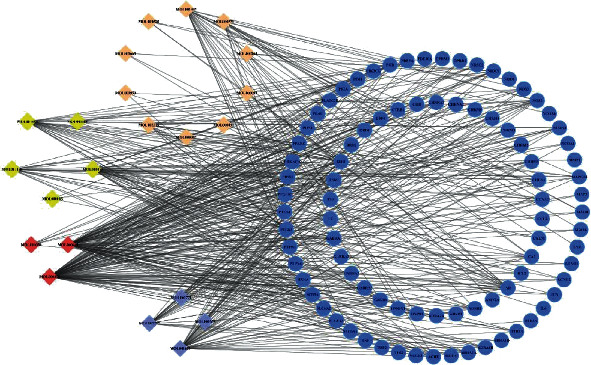
Construction of the J12 compound-putative target network. The candidate compounds of the four herbs and their putative targets were connected to construct a putative target network of compounds. The nodes representing the candidate compounds are shown as multicolored quadrilateral, and the targets are represented by blue circles.

Then, we erected a compound-target network from the selected active compounds and their potential targets to identify the complex interactions between them (Figure 3). Throughout the network, we found that most compounds are involved in the regulation of multiple targets (for example, beta-sitosterol, stigmasterol, 1,2,5,6-tetrahydrotanshinone, sugiol, and wogonin). They may be the key multieffect compounds of J12 and play a pharmacological role in OIPN and other diseases.

**Figure 4:**
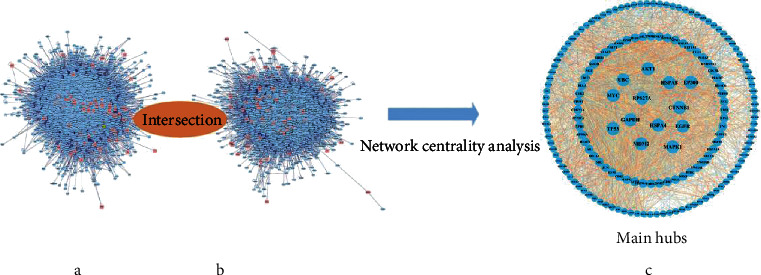
Network centrality analysis: prediction of compound targets and construction of known OIPN-related targets (a, b). Identification of advertising and screening therapeutic targets for J12 (c).

We used the screened active compounds and potential targets to construct a compound-target network to elucidate the complex interactions between them. Eighty-seven genes were gathered from five databases (GAD, PharmGKB, DrugBank, TTD, and OMIM) and recognized as CIPN-related targets. Genes and proteins do not act by acting independently of each other but through interconnected molecular networks and pathways acting at multiple levels [21]. Therefore, we selected proteins as nodes to generate the network (Figure 4). First, through previous screening and prediction, we constructed a protein-protein interaction (PPI) network for J12-related targets (3955 nodes and 96391 edges) (Figure 4(a)). Next, we constructed a PPI network of OIPN-related targets (3877 nodes and 88138 edges) (Figure 4(b)). We then combined the two networks to form a core protein-protein interaction (CPPI) network. To determine the important proteins in the CPPI network, the Cytoscape plugin CytoNCA was then used for concentration analysis [22]. .DC,” “BC,” “CC,” “EC,” “NC,” and “LAC” are set to medians of 80, 3398.987, 0.474, 0.0169, 20.668, and 14.984, respectively. Finally, we considered the 170 candidate targets as the main hubs (Figure 4(c)).

**Figure 5:**
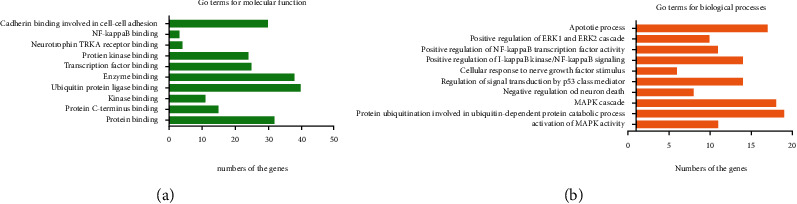
GO analysis shows representative molecular functions (a) and biological processes (b).

Through the DAVID and Metascape databases, further research on potential pathways involving candidate targets, the results were divided into two categories, namely, molecular functions/biological processes and signaling pathways. As shown in Figure 5, gene ontology (GO) analysis showed that J12 participation in the biological process ranked top: protein ubiquitination was involved, including in ubiquitin-dependent protein catabolic process, MAPK cascade, apoptotic process, positive regulation of ERK1 and ERK2 cascade, and negative regulation of neuron death. The analysis showed that the molecular functions involved were protein binding, protein kinase binding, and NF-kappaB binding.

**Figure 6:**
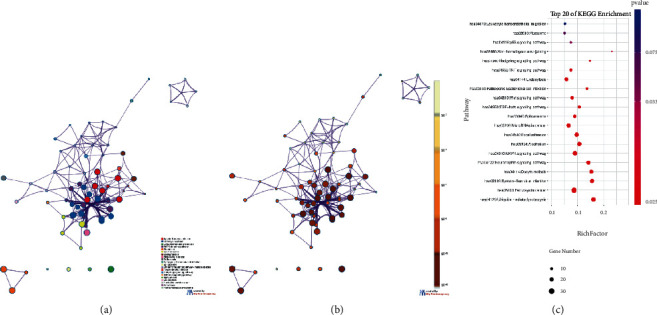
Pathway enrichment analysis: Enriched representative signal transduction pathways. Nodes in the same enriched network are colored with *p* values. The darker the color is, the more statistically important the node is. The larger the Rich factor is, the higher the enrichment degree is.

The results of KEGG annotation analysis showed that J12 participates in signaling pathways, including the MAPK signaling pathway, ubiquitin-mediated proteolysis, and neurotrophin signaling pathway (Figure 6). Based on these data, we speculate that J12 may inhibit OIPN by participating in the regulation of the MAPK pathway. Therefore, to verify the influence of J12 in the MAPK pathway, we conducted relevant molecular biological assays.

### 4.4. J12 Prevents Oxaliplatin-Induced p-ERK1/2, p-p38 and NF-*κ*B Activation in DRGs

**Figure 7:**
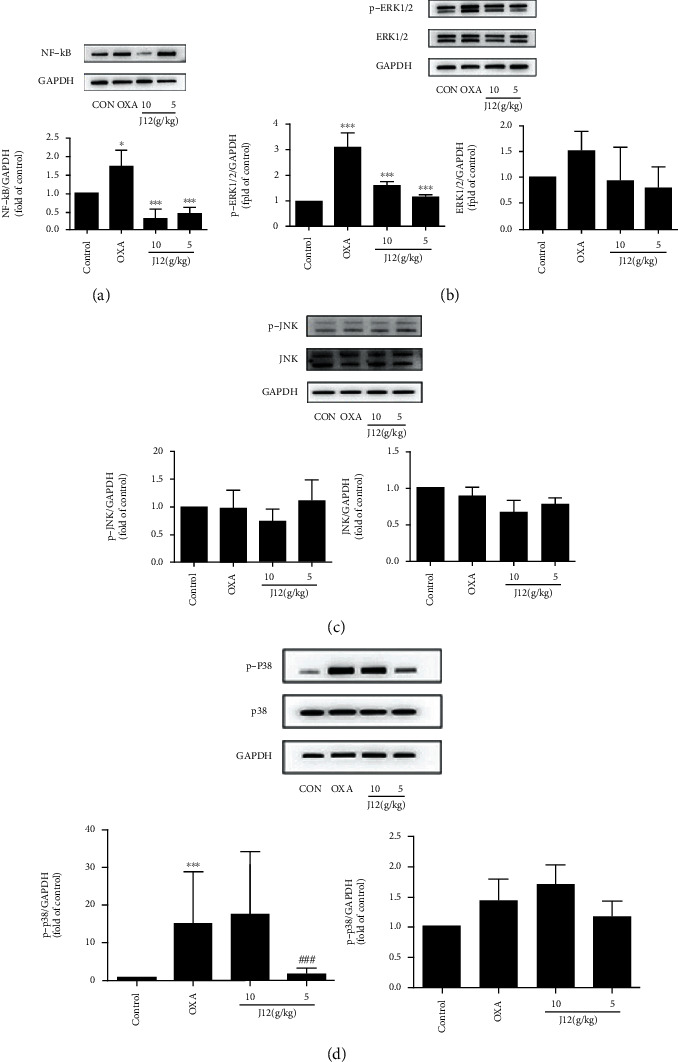
Compared with mice in the CONTROL group, p-p38, p-ERK1/2, and NF-*κ*B in the OXA group were activated. Compared with the OXA group, the same proteins in the treatment groups were mostly decreased (a, c, and d). The O+J12(L) group showed better preventive effects than the O+J12(H) group in p-p38 (d). Otherwise, the high-dose group was better than the NF-*κ*B (a) group. In conclusion, J12 prevents oxaliplatin-induced NF-*κ*B (a), p-ERK1/2 (b), and p-p38 (d) activation in the DRG. ^∗^ vs. Control, *p* < 0.05; ^∗∗^ vs. Control, *p* < 0.01; ^#^ vs. OXA, *p* < 0.05; ^##^ vs. OXA, *p* < 0.01, ^###^ vs. OXA, *p* < 0.001, *α* = 0.05.

**Figure 8:**
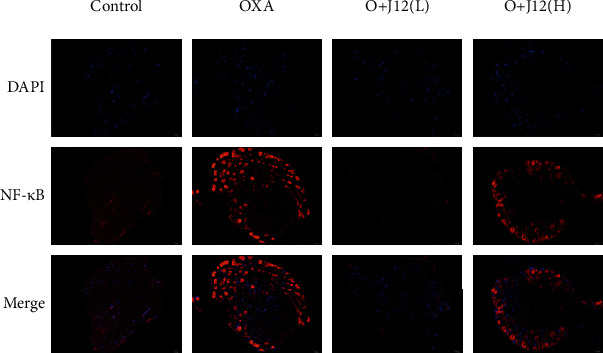
In general, the expression of NF-*κ*B in normal nerve cells is rare. From the figure, we found that the activation of NF-*κ*B was induced by OXA. The immunofluorescence results demonstrated that J12 could prevent oxaliplatin-induced NF-*κ*B activation in the DRG (scale bar = 50 *μ*m).

Based on the above analysis, to verify whether the MAPK signaling pathway and NF-*κ*B are involved in oxaliplatin-induced neuroinflammation, western blot analysis was performed to detect the protein expression levels of MAPK and NF-*κ*B in the DRG. Compared to control mice, p-p38, p-ERK1/2, and NF-*κ*B were activated in the OXA group. Compared to the OXA group, most of the same proteins were reduced in the treatment group (Figures 7(a), 7(b), and 7(d)). Immunofluorescence results also revealed the role of NF-*κ*B in oxaliplatin-induced neuropathic pain (Figure 8). These results suggest that J12 prevents oxaliplatin-induced neuropathic pain by activating p38, ERK1/2, and NF-*κ*B signaling pathways without activating p-JNK/JNK (Figure 7(c)).

### 4.5. J12 Attenuates Oxaliplatin-Induced TNF-*α*, IL-6, and IL-1*β* Activation in DRGs

**Figure 9:**
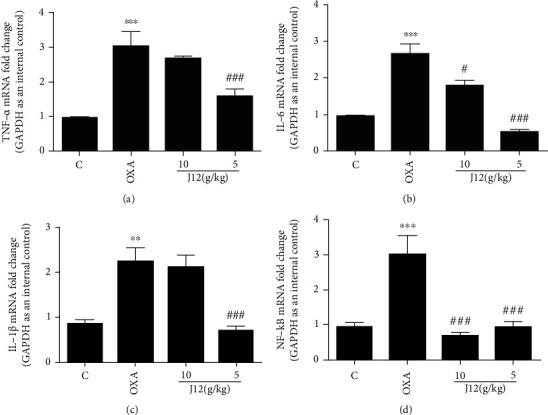
Quantitative RT-PCR showed that J12 could prevent oxaliplatin-induced TNF-*α* (a), IL-6 (b), IL-1*β* (c), and NF-*κ*B (d) activation in the DRG. ^∗^ vs. Control, *p* < 0.05; ^∗∗^ vs. Control, *p* < 0.01; ^∗∗∗^ vs. Control, *p* < 0.001; ^#^ vs. OXA, *p* < 0.05; ^##^ vs. OXA, *p* < 0.01; ^###^ vs. OXA, *p* < 0.001, *α* = 0.05.

By measuring quantitative RT-PCR, we found that the mRNA of TNF-*α*, IL-6, and IL-1*β* were activated in the OXA group comparing with control group. However, the prophylactic administration of J12 could effectively prevent these upregulations, and the preventive effect of the low-dose (5 g/kg) prophylaxis group was significantly better than that of the high-dose (10 g/kg) group (Figures 9(a), 9(b), and 9(c)).

### 4.6. J12 Prevents the Increase in Oxaliplatin-Induced Inflammatory-Related Factors in the Serum of Mice

**Figure 10:**
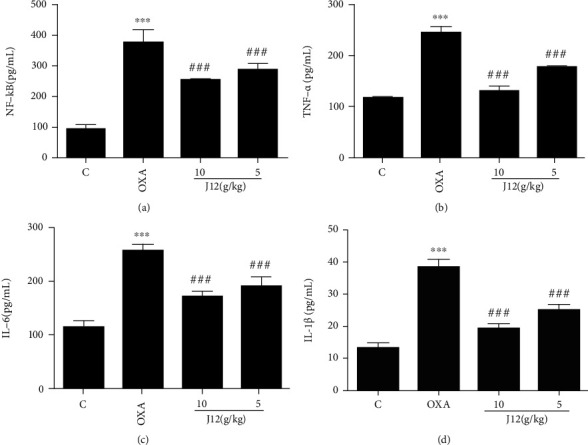
ELISA showed that J12 could prevent oxaliplatin-induced NF-*κ*B (a), TNF-*α* (b), IL-6 (c), and IL-1*β* (d) activation in the serum of mice. ^∗∗∗^ vs. Control, *p* < 0.001; ^###^ vs. OXA, *p* < 0.001, *n* = 6.

ELISA results show that compared with the control group, inflammatory-related factors of the mouse serum were significantly elevated by injecting oxaliplatin. This phenomenon was significantly improved in the preventive treatment group (Figure 10). However, it is interesting to find that in the serum, the preventive effect of the high-dose (10 g/kg) prophylaxis group was significantly better than that of the low-dose (5 g/kg) group. This is different from the results of those factors in DRG.

## 5. Discussion

Oxaliplatin is still one of the main treatments for colorectal cancers [23]. However, its anticancer efficacy is related to adverse drug reactions, especially chemotherapy-induced peripheral neuropathy (CIPN), which is the main dose-limiting toxicity of this therapy [24]. OIPN presents specific sensory disturbances, pain induced by cold and warmth, and decreased vibration perception of the hands and feet [25]. Therefore, finding a way to protect against this neuropathic pain is critical. According to an analysis of the literature, despite many neuron disease therapeutic clinical trials, no standard evidence-based treatment exists. In 2012, ASCO reported that the antidepressant duloxetine can slow down the associated numbness and tingling symptoms caused by *Taxol* or platinum but lacks systematic research evidence. However, colorectal cancer survivors now account for the third largest group of cancer survivors [26], and efficient strategies are needed to improve the prevention and/or treatment of OIPN. Therefore, we started to explore Chinese herbal medicine, which is much less toxic.

J12 has been proven to strengthen bones and tendons, to promote blood circulation, and to remove blood stasis in the clinic. Moreover, J12 is often used to treat the symptoms of weakness, pain, and edema of the lower limbs, which result from blood stasis [12]. J12 could prevent oxaliplatin-induced peripheral neuralgia based on our behavioral tests. However, there are no articles on the roles of J12 in the prevention and treatment of oxaliplatin-induced neuroinflammation.

Considering the complexity and diversity of herbal formulation components, it may be difficult to elucidate the mechanism of J12 treatment for CIPN; hence, the systematic approach was carried out first. First, we gathered a batch of active compounds in J12 by screening absorption, distribution, metabolism, and excretion (ADME). After that, the target of J12 is predicted, and a composite target network is constructed to further understand the interaction of composite targets, to further identify the key regulatory factors that these targets play as an important role in CIPN therapy. We combined two PPI networks to generate a CPPI network, one for J12 targets and the other for CIPN-related targets. By screening the topological characteristics of the CPPI network, we obtained key proteins that may contribute to CIPN therapy. Using the DAVID database and Omicshare software, the MAPK signaling pathway was identified as the main pathway.

Nuclear factor kappa light chain enhancer of activated B cells (NF-*κ*B) is a ubiquitous transcription factor well known for its role in the innate immune response. As such, NF-*κ*B is a transcriptional activator of inflammatory mediators, such as cytokines. It has been reported that downregulating NF-*κ*B can reduce inflammation [27]. Tumor necrosis factor *α* (TNF-*α*) is a master cytokine that mediates inflammatory responses and innate immunity. Guadalupe Sabio [28] has reviewed the mechanisms that mediate this dual role of MAP kinases in signal transduction mediated by TNF-*α*. It was stated that the activation of MAP kinases and TNF-*α* is synchronized. Interestingly, p38 MAP kinases can also inhibit NF-*κ*B activity following exposure to TNF-*α* [29, 30]. It has long been proven that reducing IL-6 and IL-1*β* expression can reduce inflammation [31, 32], similar to the data in this article. Another interesting finding is that in our results, it was shown that the prevention and treatment effects of J12 low-dose and high-dose groups are completely different in DRG and serum. Our hypothesis is that chemotherapy drugs affected this result in mice because chemotherapeutic drugs can accumulate in the dorsal root ganglia (DRG) [33, 34] and are considered to be used to deliver noxious stimuli [35, 36].

## 6. Conclusions

In our study, we investigated the effect of J12 on oxaliplatin-induced inflammatory pain and the potential underlying mechanisms. The results suggested that J12 had an anti-inflammatory effect in mice, inhibited by the activation of p38/NF-*κ*B and/or ERK/NF-*κ*B induced by oxaliplatin. J12 could reduce the oxaliplatin-induced production of TNF-*α*, IL-6, and IL-1*β* in the dorsal root ganglion. Overall, these data suggested that J12 may be effective in attenuating neuroinflammation in mice. These findings may aid in further exploration of the mechanism of J12 prevention of oxaliplatin-induced inflammation.

## Data Availability

The data used to support the findings of this study are available from the corresponding author upon reasonable request.

## Supplementary Materials

Supplementary Materials Figure S1: fingerprint of Siwei Jianbu Decoction and its common pattern. Taking the shared model as a reference, taking the shared model as a reference, the similarities between S1 and S10 are 0.930, 0.951, 0.937, 0.943, 0.834, 0.931, 0.936, 0.958, 0.886, and 0.919, respectively. Figure S2: fingerprint common peak and reference spectrum: 14 main common peaks can be seen in the fingerprint spectrum, accounting for more than 90% of the total peak area. Comparing the retention time with the chromatogram of the reference substance, the common peaks 4, 5, and 10 are, respectively, identified as paeoniflorin, *β*-ecdysterone, and salvianolic acid B.
