# Supplementary material for: Based on Systematic Pharmacology: Molecular Mechanism of Siwei Jianbu Decoction in Preventing Oxaliplatin-Induced Peripheral Neuropathy
Source: Neural Plast. 2020 Oct 6;2020:8880543. doi: 10.1155/2020/8880543 (PMC7559195; doi:10.1155/2020/8880543)
Supplement: Supplementary Materials — Figure S1: fingerprint of Siwei Jianbu Decoction and its common pattern. Taking the shared model as a reference, taking the shared model as a reference, the similarities between S1 and S10 are 0.930, 0.951, 0.937, 0.943, 0.834, 0.931, 0.936, 0.958, 0.886, and 0.919, respectively. Figure S2: fingerprint common peak and reference spectrum: 14 main common peaks can be seen in the fingerprint spectrum, accounting for more than 90% of the total peak area. Comparing the retention time with the chromatogram of the reference substance, the common peaks 4, 5, and 10 are, respectively, identified as paeoniflorin, β-ecdysterone, and salvianolic acid B. [file 8880543.f1.docx]

**Supplementary materials
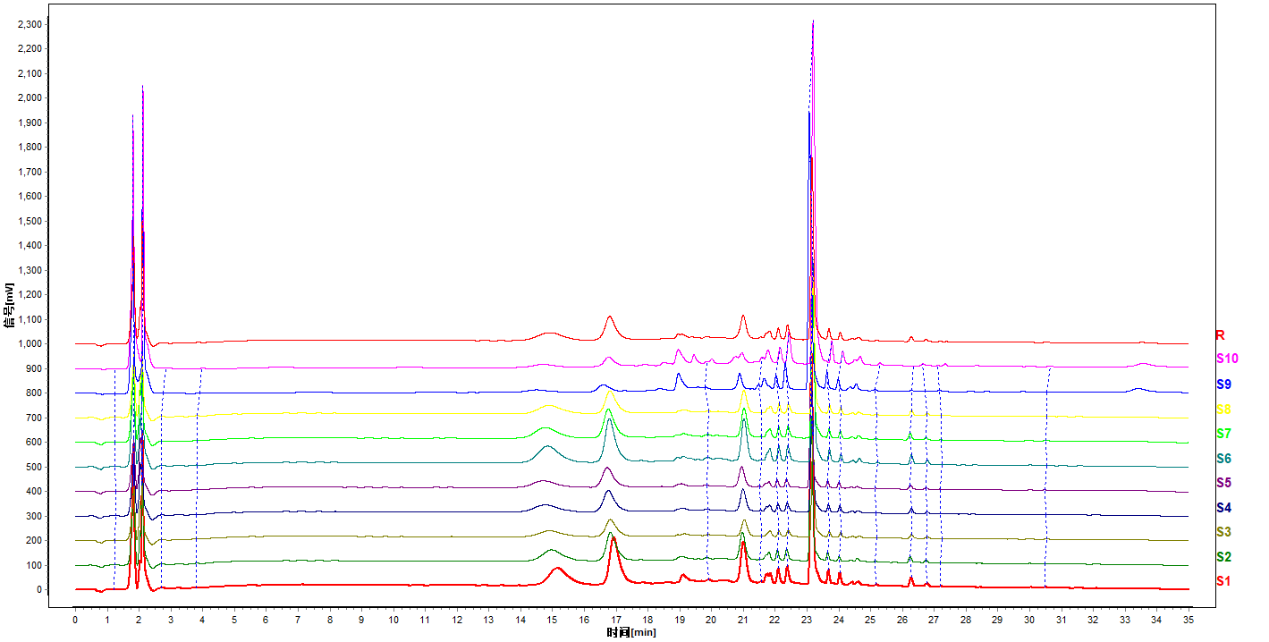
**

FIGURE S1: Fingerprint of Siwei Jianbu Decoction and its common pattern. Taking the shared model as a reference, taking the shared model as a reference, the similarities between S1 and S10 are 0.930, 0.951, 0.937, 0.943, 0.834, 0.931, 0.936, 0.958, 0.886, 0.919, respectively.

**
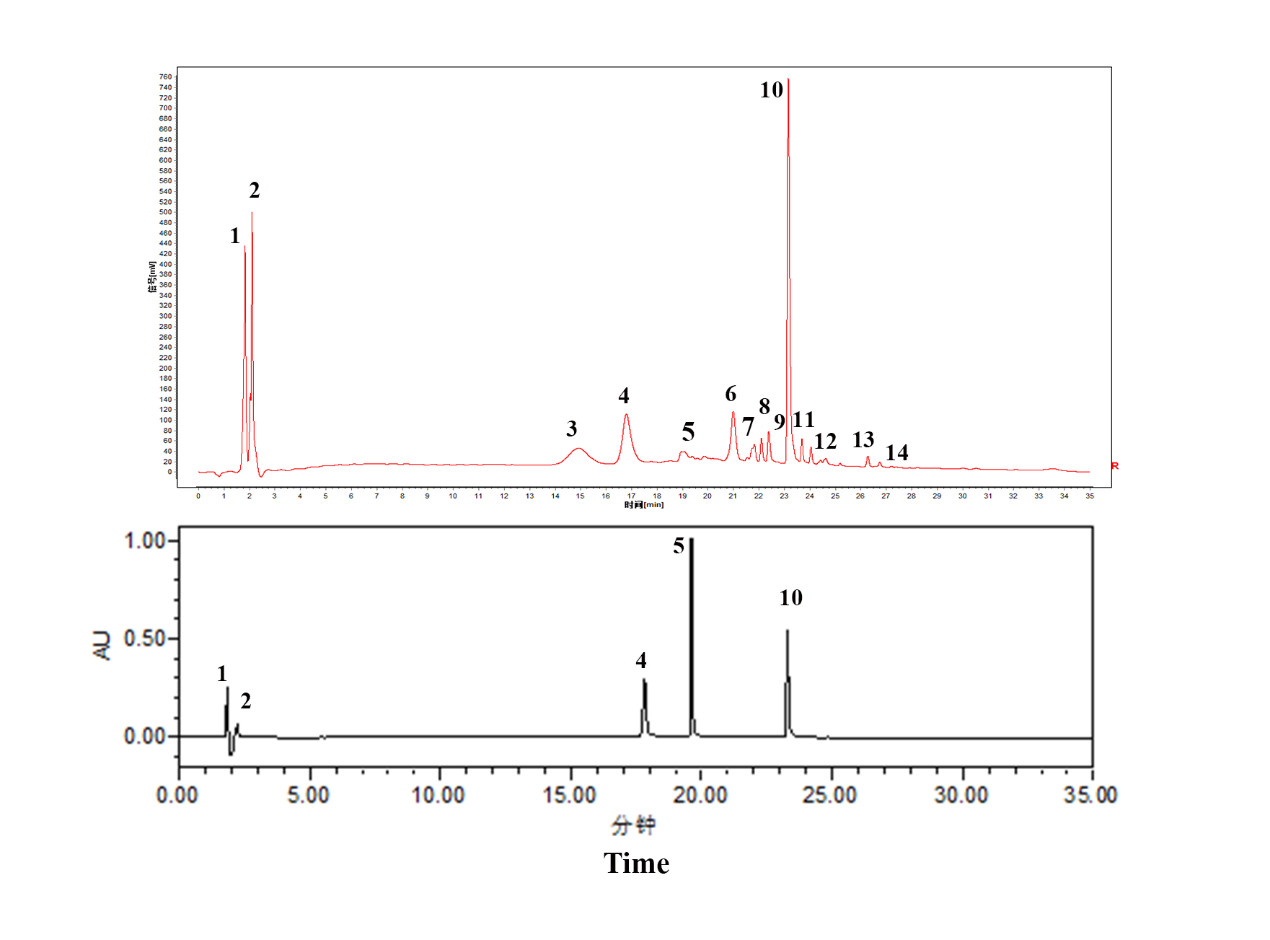
**

FIGURE S2: Fingerprint common peak and reference spectrum:14 main common peaks can be seen in the fingerprint spectrum, accounting for more than 90% of the total peak area. Comparing the retention time with the chromatogram of the reference substance, the common peaks 4, 5, and 10 are respectively identified as paeoniflorin, β-ecdysterone and salvianolic acid B.
